# Effect of Neuropeptide S Administration on Ultrasonic Vocalizations and Behaviour in Rats with Low vs. High Exploratory Activity

**DOI:** 10.3390/ph14060524

**Published:** 2021-05-30

**Authors:** Kadri Kõiv, Denis Matrov, Trine Uusen, Jaanus Harro

**Affiliations:** 1 Institute of Psychology, University of Tartu, Ravila 14A, 50411 Tartu, Estonia; Kadri.Koiv@ut.ee (K.K.); trineuusen@gmail.com (T.U.); 2 The Centre of Excellence in Neural and Behavioural Sciences, School of Natural Sciences and Health, Tallinn University, Narva Rd. 29, 10120 Tallinn, Estonia; Denis.Matrov@ut.ee; 3 Chair of Neuropsychopharmacology, Institute of Chemistry, University of Tartu, Ravila 14A, 50411 Tartu, Estonia

**Keywords:** neuropeptide S (NPS), exploratory activity, ultrasonic vocalizations (USVs), 50 kHz USVs, long and short 22 kHz USVs, individual differences

## Abstract

Neuropeptide S (NPS) is a peptide neurotransmitter that in animal studies promotes wakefulness and arousal with simultaneous anxiety reduction, in some inconsistency with results in humans. We examined the effect of NPS on rat ultrasonic vocalizations (USV) as an index of affective state and on behaviour in novel environments in rats with persistent inter-individual differences in exploratory activity. Adult male Wistar rats were categorised as of high (HE) or low (LE) exploratory activity and NPS was administered intracerebroventricularly (i.c.v.) at a dose of 1.0 nmol/5 µL, after which USVs were recorded in the home-cage and a novel standard housing cage, and behaviour evaluated in exploration/anxiety tests. NPS induced a massive production of long and short 22 kHz USVs in the home cage that continued later in the novel environment; no effect on 50 kHz USVs were found. In LE-rats, the long 22 kHz calls were emitted at lower frequencies and were louder. The effects of NPS on behaviour appeared novelty- and test-dependent. NPS had an anxiolytic-like effect in LE-rats only in the elevated zero-maze, whereas in HE-rats, locomotor activity in the zero-maze and in a novel standard cage was increased. Thus NPS appears as a psychostimulant peptide but with a complex effect on dimensions of affect.

## 1. Introduction

Neuropeptide S (NPS), a 20 amino acid peptide transmitter, has been at the forefront of neuropsychiatric peptide research since the endogenous ligand for G-protein coupled NPS receptor (NPSR), formerly known as an orphan receptor GPR154, was identified almost 20 years ago by Reinscheid and colleagues [1]. NPS and its receptors are found in several tissues, with the highest expression in the thyroid, salivary and mammary glands, and the brain [1]. In the brain, NPS is mainly expressed in a few pontine cell clusters, while NPSR is widely distributed, including regions of the hypothalamus, thalamus, cortex, and amygdala [1,2] that are implicated in the regulation of, e.g., homeostasis, sensory gating, and emotional processing. Accordingly, NPS modulates wakefulness, arousal, feeding, memory, and anxiety-like states in rodents. Intracerebroventricular (i.c.v.) administration of NPS elicits a unique behavioural profile of increased wakefulness and arousal along with anxiolytic-like effects at similar doses [1,3]. NPS has robustly increased locomotor activity in mice and rats both in novel and habituated surroundings and anti-anxiety effects have been reported in a variety of behavioural tests [3,4,5,6,7].

Research of NPS in humans revealed the significance of a functional polymorphism (rs324981 A > T) in the gene of the NPS receptor, *NPSR1*, to the potency of receptor-bound NPS [8]. The T-allele encoding receptor protein with higher signal transduction efficacy has been associated with heightened autonomic arousal and with panic disorder [9] and with an increased response of the amygdala to fearful stimuli [10]. This seems contradictory to the anxiolytic effects of NPS in animal studies. In contrast, studies in a representative birth cohort sample instead associated the A-allele of *NPSR1* rs324981, responsible for the less efficient receptor, with higher anxiety, but the T-allele with higher impulsivity [11,12]. These findings suggest that the action of NPS may depend on individual differences and environmental contingencies. Indeed, the NPS system seems to have played a role in shaping the adaptive responses to the environment since the vertebrate water-to-land transition [13], and the less efficient A-allele of the *NPSR1* rs324981 is thought to be derived from the ancestral more efficient T-allele [14]. NPS is endogenously released in response to acute stressors [15], and its administration activates the hypothalamo-pituitary-adrenal (HPA) stress axis [16,17]. NPS neurotransmission interacts with several neurochemical systems in modulating arousal and locomotion, including, among others, corticotropin-releasing factor (CRF) and dopamine (DA) systems [2,18,19,20].

While the readouts of many animal models of anxiety can be severely confounded by aspects of novelty and other factors shaping the approach-avoidance conflict [21], ultrasonic vocalization is a more direct window into the affective state of mammals [22]. Communication in the highly social *Rattus norvegicus* is carried out in the range of ultrasonic frequencies above 20 kHz, and ultrasonic vocalizations (USVs) emitted are thought to communicate emotional states to conspecifics in a variety of aversive and appetitive behavioural situations [23,24,25]. Two main types of adult rat USVs have been described: longer-lasting lower frequency 22 kHz calls which are emitted by rats subjected to negative/aversive conditions, such as exposure to acute stressors (foot-shock and air-puff), a predator, social defeat, negative drug experience, etc. [26,27,28,29]; and higher frequency 50 kHz calls of shorter duration, which are brought about in the context of positive/appetitive states, such as rough-and-tumble play, seeking for a reward, tickling, and administration of psychostimulants [30,31,32]. Furthermore, subtypes of both classes of USVs have been identified; the 22 kHz calls can further be divided into short (duration up to 300 ms) and long (over 300 ms calls) forms [33], both of which are associated with aversive states. Fifty kHz USVs comprise three biologically significant subtypes: flat, frequency-modulated, and trill calls (reviewed in [34]), all expressing positive emotional states.

Two separate brain systems are responsible for eliciting 22 kHz and 50 kHz vocalizations (for review, see [23]). The 22 kHz USV production is related to signalling in the ascending cholinergic fibers that originate from the laterodorsal tegmental nucleus [35], while 50 kHz USVs are dependent on the mesolimbic dopaminergic circuitry that comprises axon projections from the ventral tegmental area (VTA) to the nucleus accumbens (NAc) [32,36]. Twenty-two kHz calls can be pharmacologically induced by direct cholinergic stimulation of the brain [37] as well as by application of ligands with aversive effects, e.g., naloxone [27]; agents that potentiate mesolimbic dopaminergic neurotransmission concurrently also increase 50 kHz USVs [32,38,39].

Studying inter-individual differences in depressed and anxious phenotypes at behavioural, physiological, and genetic levels helps in revealing the substrates for vulnerability to depression and clarifying its pathogenetic mechanisms [40,41]. Two of the depressive features, namely reduced motivation (to explore) and heightened anxiety, can be jointly studied in terms of novelty-related, exploratory behaviour. Conflicting motivations —an exploratory drive (curiosity) and a fear evoked by novelty—are simultaneously shaping exploration, that in some conditions leads to non-Gaussian distribution of behaviour [42]. We have developed and pharmacologically characterised a test of spontaneous exploratory activity [43], the exploration box test, which combines features of both forced and free exploration and separates rats into clusters with persistently low motivation to explore/high anxiety, and high motivation to explore/low anxiety (low explorers—LE, and high explorers—HE, respectively). LE- and HE-rats display profoundly different adaptive strategies in this test, and it is predictive of their further novelty-, anxiety-, and depression-related behaviours [44]. 

As compared to the HE-rats, the behaviourally ‘depressive-like’ LE-rats also behave more anxiously in the elevated plus-maze, display more passive coping strategies in the forced swimming test and retain a more enduring association between neutral and stressful stimuli in the fear conditioning test [44]. Multiple neurochemical differences may underlie the behavioural variation observed in LE- and HE-rats, from differently expressed depression-related genes in raphe nuclei, hippocampus and frontal cortex [45] to neurochemical differences in all three major monoaminergic systems [46], especially in striatal dopaminergic neurotransmission [44,47]. Furthermore, LE-rats are less sensitive to the acute and chronic effects of antidepressants on amphetamine-stimulated striatal dopamine and serotonin release [48].

Thus, based on animal and human studies it can be hypothesised that NPS receptor stimulation has an effect on primary affective processes that may differ between more and less anxious individuals. Ultrasonic vocalizations at 22 kHz and 50 kHz offer the most direct indicator of affect in rodents, reflecting what is in humans interpreted as negative and positive emotion, respectively. With this background, the aim of the present study was to examine the effect of NPS on USVs and to find out whether the LE- and HE-rats would differ in their sensitivity to intracerebroventricular NPS treatment. We hypothesised that NPS would elicit 50 kHz USVs and increase activity in HE-rats, especially in novel environments, but in LE-rats 50 kHz would be produced only in more familiar contexts and that distress vocalizations may be induced, especially in novel surroundings. We further explored whether NPS would alter the acoustic features of USVs, and whether it would be dependent on the exploratory phenotype of the rats.

## 2. Results

### 2.1. Effect of Intracerebroventricular Administration of NPS on Ultrasonic Vocalizations

**Figure 1:**
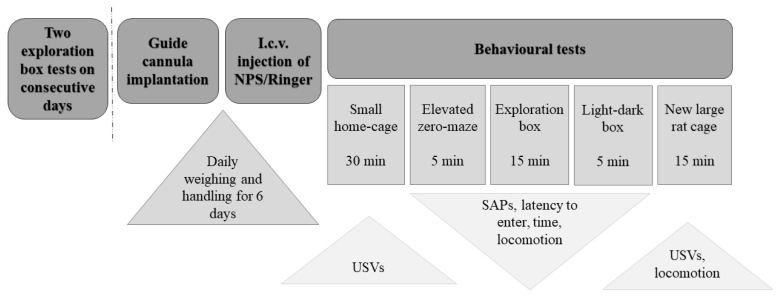
The general timeline of experimental procedures. I.c.v.: intracerebroventricular; NPS: neuropeptide S; SAPs: stretch-attend postures; USVs: ultrasonic vocalizations.

Ultrasonic vocalizations were recorded for 30 min in the home-cage after i.c.v. NPS or vehicle administration (Figure 1). 

**Figure 2:**
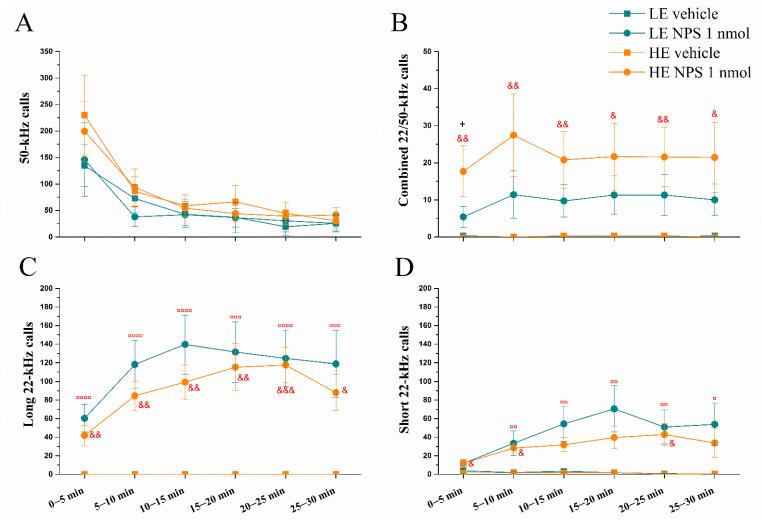
Number of ultrasonic vocalizations emitted in the rat’s home-cage after administration of vehicle/NPS (1 nmol, i.c.v.): 50 kHz (**A**), combined 22/50 kHz (**B**), long 22 kHz (**C**) and short 22 kHz (**D**) calls (data shown as mean ± SEM). &, &&, &&&: *p* < 0.05, 0.01, 0.001 vs. HE vehicle, respectively; ¤, ¤¤, ¤¤¤, ¤¤¤¤: *p* < 0.05, 0.01, 0.001, 0.0001 vs. LE vehicle, respectively; +: *p* < 0.05 vs. LE NPS. Red colour denotes difference from respective vehicle group. HE: high exploring rats; LE: low exploring rats; NPS: neuropeptide S. HE vehicle (*n* = 7); HE NPS (*n* = 9); LE vehicle (*n* = 10); LE NPS (*n* = 10).

The overall number of 50 kHz calls was most abundant during the first five minutes (Figure 2) and diminished over the 30 min period (repeated measures effect of *Time* F(5,160) = 23.4, *p* < 0.0001). Administration of NPS did not affect the level of expression of 50 kHz calls either in LE- or HE-rats (Figure 2A). However, central NPS administration elicited a massive production of long and short 22 kHz USVs, and also calls with both 22/50 kHz components (effect of *NPS* F(1,32) = 39.2, *p* < 0.0001; F(1,32) = 14.0, *p* < 0.001; F(1,32) = 11.3, *p* < 0.01, respectively; Figure 2B–D, respectively). Only the NPS-treated animals elicited long 22 kHz calls (all of the NPS-treated rats; LE = 9 and HE = 10). These started on average 170 s after administration and did not differ in amount between HE- and LE-rats. The number of long and short 22 kHz calls varied during the 30 min measurement period, peaking at 10–20 min after NPS administration (*Time* effect F(5,160) = 6.9, *p* < 0.0001; F(5,160) = 5.0, *p* < 0.001; interaction of *Time* × *NPS* F(5,160) = 6.9, *p* < 0.0001 and F(5,160) = 5.7, *p* < 0.0001, respectively). NPS also reduced the onsets to the first short 22 kHz call (*NPS* F(1,28) = 5.5, *p* < 0.05) and combined 22/50 kHz call (*NPS* F(1,16) = 12.1, *p* < 0.01) but did not modify the beginning of 50 kHz calls (data not shown). 

### 2.2. Acoustic Characteristics of the NPS-Elicited 22 kHz Calls in HE- and LE-Rats

In order to analyse whether the acoustic characteristics [39,49] of NPS-induced long and short 22 kHz USVs would differ in HE- and LE-rats during the 30 min period studied, duration, average peak frequency, and average peak amplitude of the USVs were each modelled as predicted by the exploratory phenotype and time-point using two modelling frameworks: linear mixed-effects models (LMM) and generalised additive models (GAM). In both modelling frameworks, random effects were used to account for each animal’s behaviour in addition to the group membership. This was necessary because the number of USVs differed between rats and repeated USVs of the same animal were correlated. In LMM, the effect of time on USV parameters was modelled parametrically as the sum of the linear, quadratic (parabolic), and cubic (hyperbolic) effects. This approach allowed us to test whether changes in the USV properties over time follow a particular well-defined geometric curve. In contrast, the GAM approach modelled the effect of time by a single nonparametric spline. It can be considered as a nonparametric ANOVA approach and is, therefore, more permissive in identifying the time-dependent effect of phenotype on USVs.

#### 2.2.1. Duration of Calls

**Figure 3:**
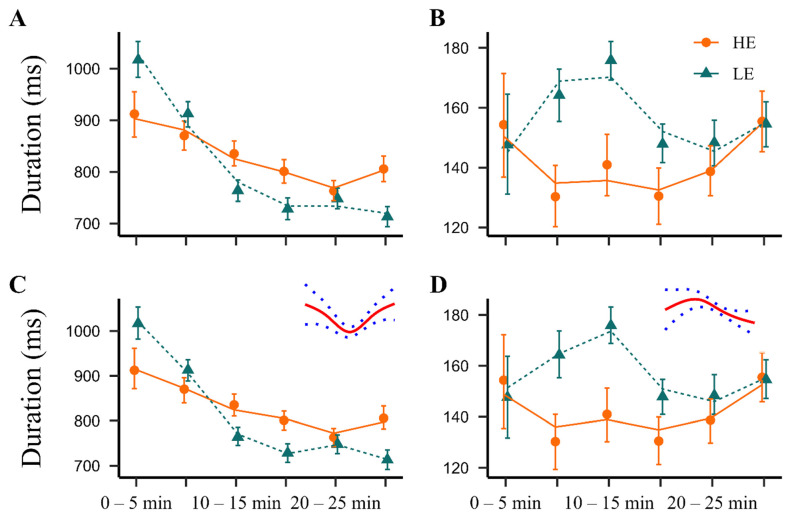
Duration of NPS-induced (1 nmol, i.c.v.) 22 kHz calls. Symbols: Observed time-binned mean durations of long (**A**,**C**) and short (**B**,**D**) 22 kHz USVs (error bars indicate 95% confidence limits for the population mean obtained by nonparametric bootstrap). Lines: Model-based predictions from linear mixed-effects models (LMM) (**A**,**B**) and generalised additive models (GAM) (**C**,**D**; difference in splines added). HE: high exploring rats; LE: low exploring rats; NPS: neuropeptide S. HE NPS (*n* = 9); LE NPS (*n* = 10).

Figure 3 presents how model-based predictions fit the obtained data on duration of calls. Generally, the longest 22 kHz calls were emitted during the first five minutes after i.c.v. NPS (Figure 3A,C), after which the duration of the long 22 kHz calls gradually decreased. No difference between LE- and HE-rats was found for both short and long 22 kHz USVs by linear mixed-effects models. In contrast, modelling the time-dependent change in the duration of USVs by cubic splines found the difference between phenotypes to be significant both for short (*p* < 0.05) and long 22 kHz USVs (*p* < 0.001). However, combining the results from the two modelling frameworks, the duration of 22 kHz calls did not differ between the LE- and HE-phenotypes.

#### 2.2.2. Average Peak Frequency

**Figure 4:**
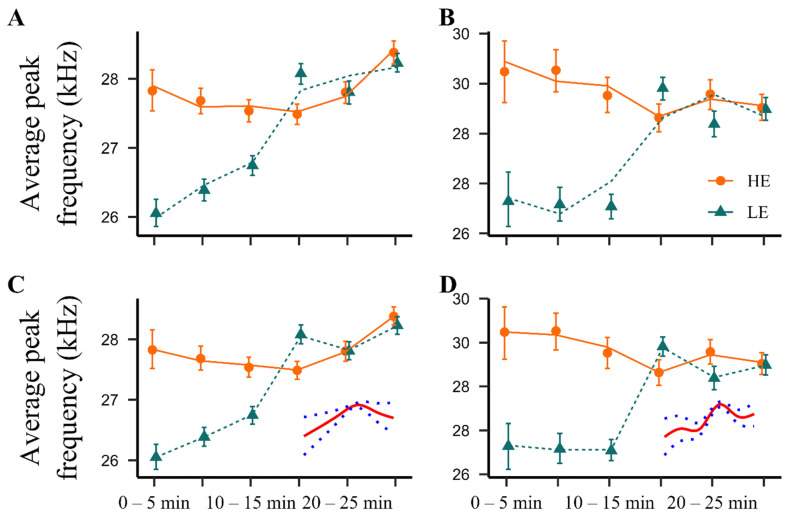
Average peak frequency of NPS-induced (1 nmol, i.c.v.) 22 kHz calls. Symbols: Observed time-binned average peak frequencies of long (**A**,**C**) and short (**B**,**D**) 22 kHz USVs (error bars indicate 95% confidence limits for the population mean obtained by nonparametric bootstrap). Lines: Model-based predictions from LMM (**A**,**B**) and GAM (**C**,**D**; difference in splines added). HE: high exploring rats; LE: low exploring rats; NPS: neuropeptide S. HE NPS (*n* = 9); LE NPS (*n* = 10).

For short 22 kHz USVs, the LMM modelling framework identified no difference between LE- and HE-rats on average peak frequency of calls (Figure 4B). However, for long 22 kHz USVs a significant difference was found between parabolic shapes of time-dependent curves in the two phenotypes (*p* < 0.05; Figure 4A). Furthermore, when using the GAM approach, modelling the time-dependent curves by cubic splines indicated the difference between phenotypes to be highly significant for both types of 22 kHz USVs (*p* < 0.001, Figure 4C,D). Taken together, during the first 15 min after the i.c.v. injection of NPS, the long 22 kHz USVs emitted by the LE-rats were at significantly lower frequencies than those emitted by the HE-rats. 

#### 2.2.3. Average Peak Amplitude

**Figure 5:**
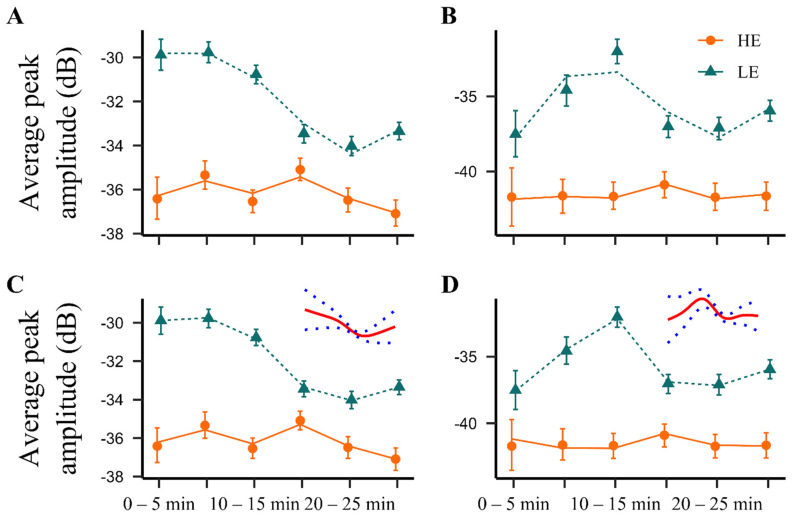
Average peak amplitude of NPS-induced (1 nmol, i.c.v.) 22 kHz calls. Symbols: Observed time-binned average peak amplitudes of long (**A**,**C**) and short (**B**,**D**) 22 kHz USVs (error bars indicate 95% confidence limits for the population mean obtained by nonparametric bootstrap). Lines: Model-based predictions from LMM (**A**,**B**) and GAM (**C**,**D**; difference in splines added). HE: high exploring rats; LE: low exploring rats; NPS: neuropeptide S. HE NPS (*n* = 9); LE NPS (*n* = 10). Please note that average peak amplitude (acoustic intensity measured in decibels (dB)) is a negative value, and a less negative value reflects a louder vocalization [50].

As with average peak frequencies, the LMM-approach found no difference in average peak amplitude between LE- and HE-rats for short USVs and a significant difference was found for long 22 kHz USVs between parabolic shapes of time-dependent curves in the two phenotypes (*p* < 0.05, Figure 5A,B). The GAM approach identified the difference between phenotypes to be highly significant for both types of 22 kHz USVs (*p* < 0.001). All in all, long 22 kHz USVs of the LE-rats were louder than in the HE-rats, especially so during the first 15 min after NPS administration.

The results from the LMM and GAM modelling show that the time-dependent changes in USV properties were non-linear. For average peak frequencies and amplitudes in long calls, the effect of the phenotype could be approximated by the parabolic curve, but for short USVs and call durations, the effect of the phenotype was both non-linear and nonparametric. The fit of model-derived predictions to the actual observations was tighter in the GAM approach.

### 2.3. Behavioural Tests

#### 2.3.1. Exploration Box Test

**Figure 6:**
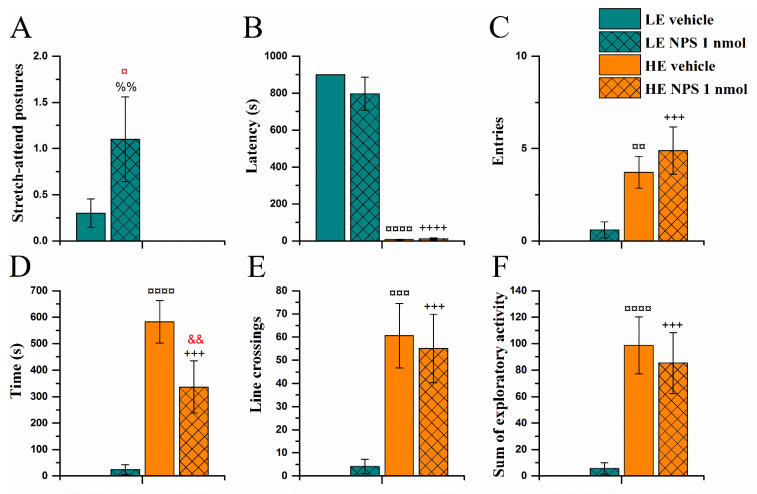
Behaviour in the exploration box test (EBT) approximately 40 min after vehicle/NPS (1 nmol, i.c.v.) administration: strech-attend postures (**A**), latency to enter (**B**), entries into (**C**), time spent, (**D**), line crossings (**E**), and sum of exploratory activity (**F**) in the open part of the EBT apparatus (data shown as mean ± SEM). &&: *p* < 0.01 vs. HE vehicle; ¤, ¤¤, ¤¤¤, ¤¤¤¤: *p* < 0.05, 0.01, 0.001, 0.0001 vs. LE vehicle, respectively; %%: *p* < 0.01 vs. HE NPS; +++, ++++: *p* < 0.001, 0.0001 vs. LE NPS, respectively. Red colour denotes difference from respective vehicle group. HE: high exploring rats; LE: low exploring rats; NPS: neuropeptide S. HE vehicle (*n* = 7); HE NPS (*n* = 9); LE vehicle (*n* = 10); LE NPS (*n* = 10).

The overall higher activity of the HE-rats in the exploration box, observed in the selection test day, was also apparent on the seventh day after the surgery, about 40 min after the central NPS/vehicle treatment (Figure 6). HE-rats entered the open area faster and more often (*Activity* effect on latency (F(1,32) = 280 and entries (F(1,32) = 27.1, both *p* < 0.0001; Figure 6B,C), and spent more time in it (F(1,32) = 56.3, *p* < 0.0001; Figure 6D). HE-rats moved around more and explored the objects in the open area more intensively (*Activity* effect on line crossings (Figure 6E), rearings, objects, and the sum of exploratory activity (Figure 6F): (F(1,32) = 35.6, F(1, 32) = 25.8, F(1, 32) = 41.7, F(1,32) = 38.1, with all *p* < 0.0001, respectively)).

NPS modified two parameters differently in HE and LE animals in the exploration box test. First, NPS shortened the time that HE-rats spent in the open area exploring (interaction of *Activity* × *NPS* for time (F(1,32) = 5.1, *p* < 0.05) while not significantly reducing their locomotor activity or objects explored. LE-rats treated with NPS also made more stretch-attend postures (SAP) towards the open arena (*Activity* effect on number of SAPs (F(1,32) = 6.6, *p* < 0.05; Figure 6A) as compared to vehicle treated controls and HE-rats, but did not enter the open area to explore more than their controls.

#### 2.3.2. Elevated Zero-Maze Test

**Figure 7:**
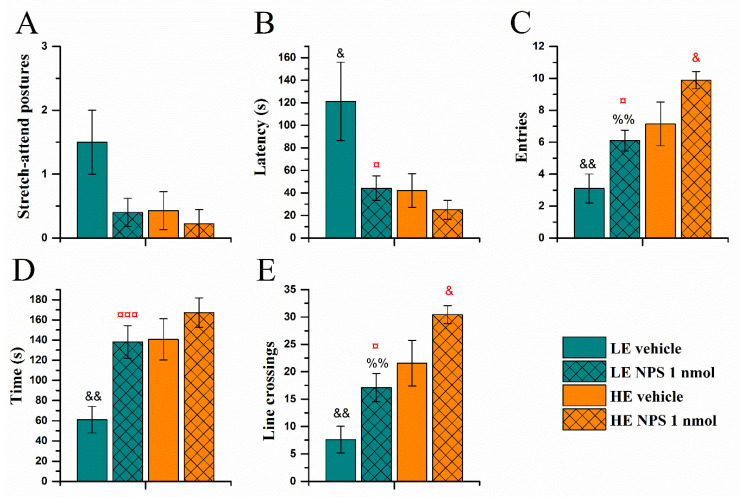
Behaviour in the elevated zero-maze approximately 35 min after vehicle/NPS (1 nmol, i.c.v.) administration: strech-attend postures (**A**), latency to enter (**B**), entries into (**C**), time spent, (**D**), and line crossings (**E**) in the open quadrants of the apparatus (data shown as mean ± SEM). &, &&: *p* < 0.05, 0.01 vs. HE vehicle, respectively; ¤, ¤¤¤: *p* < 0.05, 0.001 vs. LE vehicle, respectively; %%: *p* < 0.01 vs. HE NPS. Red colour denotes difference from respective vehicle group. HE: high exploring rats; LE: low exploring rats; NPS: neuropeptide S. HE vehicle (*n* = 7); HE NPS (*n* = 9); LE vehicle (*n* = 10); LE NPS (*n* = 10).

Similarly to the exploration box test, the HE-rats were more active in the open parts of the zero-maze (Figure 7). HE-rats entered the open quadrants faster and more often (*Activity* effect on latency (F(1,32) = 5.1, *p* < 0.05; Figure 7B) and entries (F(1,32) = 20.2, *p* < 0.0001; Figure 7C), and spent more time in the open quadrants (F(1,32) = 11,6, *p* < 0.01; Figure 7D) moving around more than the LE-rats (*Activity* effect on line crossings F(1,32) = 25.3, *p* < 0.0001; Figure 7E). 

Central administration of NPS reduced the latency to enter the open quadrant (*NPS* effect F(1,32) = 4.7, *p* < 0.05), increased the number of entries into the open quadrants (F(1,32) = 10.9, *p* < 0.01), time spent in the open quadrants (F(1,32) = 10.5, *p* < 0.01), and the number of line crossings (F(1,32) = 11.5, *p* < 0.01). *Post hoc* testing revealed that while NPS significantly increased the number of entries and line crossings both in HE- and LE-rats, the latency to enter an open quadrant was significantly decreased, and time spent in the open parts of the maze was significantly increased only in LE-rats. 

#### 2.3.3. Light-Dark Box Test

**Figure 8:**
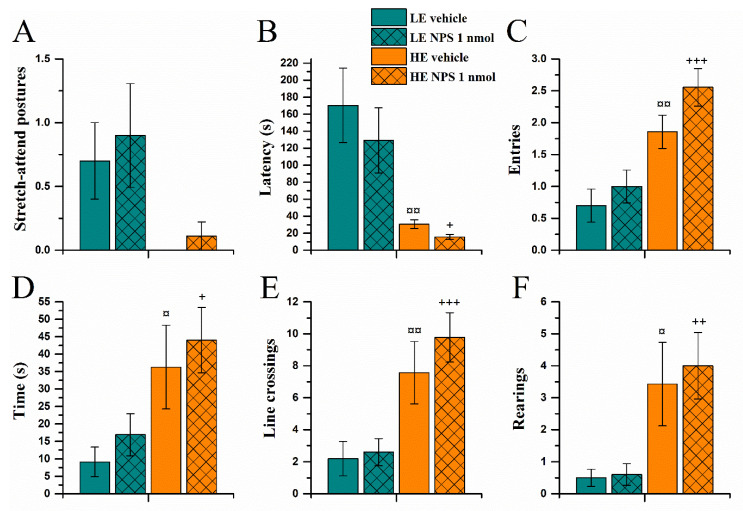
Behaviour in the light-dark box approximately 55 min after vehicle/NPS (1 nmol, i.c.v.) administration: strech-attend postures (**A**), latency to enter (**B**), entries into (**C**), time spent, (**D**), line crossings (**E**), and rearings in the light part of the apparatus (data shown as mean ± SEM). (**F**) ¤, ¤¤: *p* < 0.05, 0.01 vs. LE vehicle, respectively; +, ++, +++: *p* < 0.05, 0.01, 0.001 vs. LE NPS, respectively. HE: high exploring rats; LE: low exploring rats; NPS: neuropeptide S. HE vehicle (*n* = 7); HE NPS (*n* = 9); LE vehicle (*n* = 10); LE NPS (*n* = 10).

As in the exploration box and elevated zero-maze tests, the HE-rats were more active in the light part of the light-dark box (Figure 8). HE-rats entered the light part faster and more often (*Activity* effect on latency (F(1,32) = 14.8, *p* < 0.001, Figure 8B) and entries (F(1,32) = 24.5, *p* < 0.0001; Figure 8C), and spent more time in the light part (F(1,32) = 12.0, *p* < 0.01, Figure 8D) while also moving around more than the LE-rats (*Activity* effect on line crossings F(1,32) = 22.2, *p* < 0.0001 and rearings F(1,32) = 16.9, *p* < 0.001; Figure 8E,F). LE-rats made more SAPs towards the light compartment (*Activity* effect on SAPs F(1,32) = 6.5, *p* < 0.05; Figure 8A). 

NPS failed to modify behavior in the light-dark box test, although it tended to increase the number of entries into the light compartment (*NPS* (F(1,32) = 3.3, *p* = 0.078). 

#### 2.3.4. Novel Large Rat Housing Cage

In the last behavioural test conducted approximately 1 h after the i.c.v. injection, the rats were placed in an unfamiliar standard housing cage where their locomotion and USVs were recorded and later scored for 15 min.

##### Locomotor Activity

**Figure 9:**
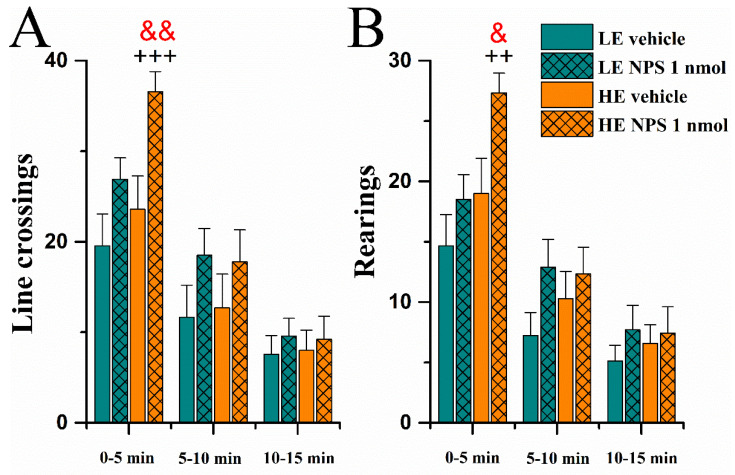
Locomotor activity in a novel large rat cage approximately 1 h after vehicle /NPS (1 nmol, i.c.v.) administration: line crossings (**A**) and rearings (**B**) (data shown as mean ± SEM). &, &&: *p* < 0.05, 0.01 vs. HE vehicle, respectively; ++, +++: *p* < 0.01, 0.001 vs. LE NPS, respectively. Red colour denotes difference from respective vehicle group. HE: high exploring rats; LE: low exploring rats; NPS: neuropeptide S. HE vehicle (*n* = 7); HE NPS (*n* = 9); LE R vehicle (*n* = 10); LE NPS (*n* = 10).

In the novel standard rat housing cage, the HE- and LE-rats did not differ in their locomotion. NPS increased both line crossings and rearings during the testing period (effect of *NPS* (F(1,31) = 5.8, *p* < 0.05 and (F(1,31) = 4.9, *p* < 0.05, respectively; Figure 9A and 9B, respectively). However, *post hoc* tests failed to show the difference between NPS and vehicle-treated HE and LE groups.

When the data were analysed in three bins of 5 min of duration each, the repeated measures ANOVA showed that rats of all groups were most active during the first five minutes and their locomotion decreased throughout the trial (*Time* effect F(2,62) = 87.4 and F(2,62) = 82.8, *p* < 0.0001 on line crossings and rearings, respectively). Furthermore, it became apparent that the effect of NPS on locomotion was dependent on time bin as well as the exploratory phenotype, as *Time* × *Activity* (F(2,62) = 4.0, *p* < 0.05) and *Time* × *NPS* interactions (F(2,62) = 4.8, *p* < 0.05) were found to be significant for line crossings and *Time* × *Activity* interaction (F(2,62) = 4.9, *p* < 0.05) for rearings. *Post hoc* tests clarified that these interactions were primarily due to a significantly higher number of line crossings and rearings in the HE/NPS group during the first 5 min of the trial. 

##### Number of USVs

**Figure 10:**
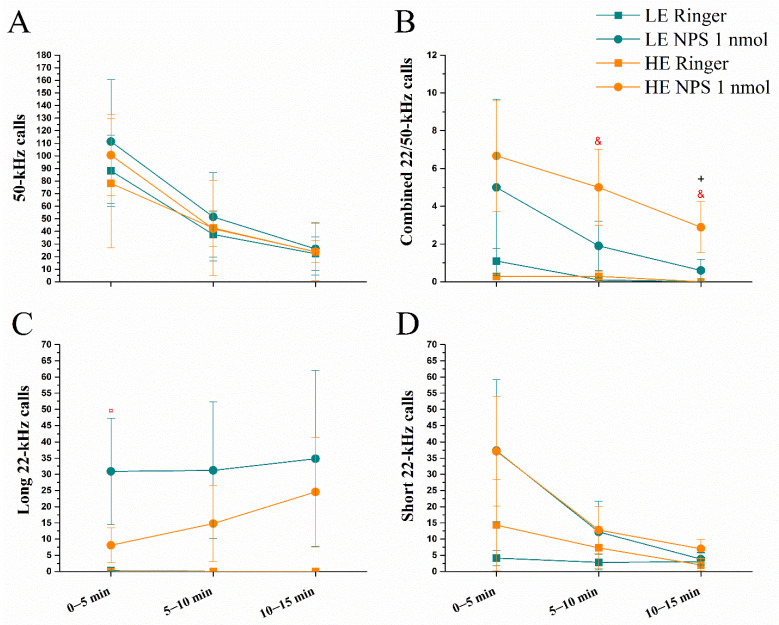
Number of ultrasonic vocalizations emitted in a novel large animal cage approximately 1 h after vehicle /NPS (1 nmol, i.c.v.) administration: 50 kHz (**A**), combined 22/50 kHz (**B**), long 22 kHz (**C**) and short 22 kHz (**D**) calls (data shown as mean ± SEM). &: *p* < 0.05 vs. HE vehicle; ¤: *p* < 0.05 vs. LE vehicle; +: *p* < 0.05 LE NPS. Red colour denotes difference from respective vehicle group. HE: high exploring rats; LE: low exploring rats; NPS: neuropeptide S. HE vehicle (*n* = 7); HE NPS (*n* = 9); LE vehicle (*n* = 10); LE NPS (*n* = 10).

As with locomotor activity, 50 kHz calls and short 22 kHz calls were most abundant during the first 5 min of the trial and then reduced in number (repeated measures *Time* effect F(2,64) = 23.2, *p* < 0.0001 and F(2,64) = 5.9, *p* < 0.01, respectively; Figure 10A,D). 

Long 22 kHz calls were emitted only by seven NPS rats, three of which were LE and four were HE-rats (*NPS* effect F(1,32) = 4.5, *p* < 0.05; Figure 10C). NPS administration also brought about more combined 22/50 kHz calls (*NPS* F(1,32) = 5.0, *p* < 0.05; Figure 10B). 

##### Correlation of Locomotor Activity and USVs

The more the rats moved around in the standard housing cage, the more 50 kHz calls were emitted during the 15 min period (correlation with line crossings and rearings (*n* = 35), r = 0.44 and 0.46, *p* < 0.01, respectively). This was largely dependent on controls as their 50 kHz vocalizations correlated with line crossings and rearings (*n* = 16, r = 0.53 and 0.50, *p* < 0.05) while in NPS-treated rats, only a negative correlation between long 22 kHz calls and locomotor activity was found (*n* = 19, line crossings r = −0.55 and rearings r = −0.48, *p* < 0.05, respectively). Thus, the more of the long 22 kHz vocalizations the NPS-treated rats elicited, the less they moved around.

## 3. Discussion

The present study demonstrated that in Wistar rats, administration of i.c.v. NPS elicits long and short 22 kHz ultrasonic vocalizations that are thought to signal a negative affective state, and that the effect of NPS on behaviour may depend on the exploratory phenotype/anxiety levels of the animal tested. To the best of our knowledge, this is the first description of an abundant production of long and short 22 kHz calls, as well as mixed USVs with both 22 and 50 kHz components, after i.c.v. NPS administration. All findings of this study are summarised in Table 1.

USVs can be used to assess both acute and conditioned drug effects, and the 22 kHz USVs provide the best index of the aversive effects of a drug [27]. As NPS was reported to provide an anxiety-relieving profile in rodents, the emergence of 22 kHz USVs, thought to signal anxiety and negative emotional state, is surprising, as was the inability of NPS treatment to modify the 50 kHz USVs that were elicited, as expected, by change of environment. Nevertheless, NPS was demonstrated to stimulate the HPA-axis, causing an increase in plasma adrenocorticotropic hormone (ACTH) levels 10 min after i.c.v. injection and plasma corticosterone 40 min after i.c.v. injection [16]. In another study, 10 nmol of NPS i.c.v. increased plasma corticosterone levels in wild-type but not in NPSR1-KO mice 40 min after administration [51]. I.c.v. injection of NPS also increased the peripheral blood concentration of adrenaline (approx. 3-fold at 15 min) and corticosterone [5]. Thus, NPS receptor stimulation potently activates the stress axis. On the other hand, administration of corticosterone alone has been shown not to elicit the emission of 22 kHz USVs or to modify the number of 50 kHz USVs produced [52], so the effect of NPS should be directly on the anxiety circuits.

NPS stimulates the release of CRF and arginine vasopressin (AVP) from hypothalamic explants, thus NPS stimulates the HPA-axis via the release of CRF and AVP [16]. Many of the NPS-positive neurons in the lateral parabrachial nucleus also co-express CRF [2]. CRF-system has been shown to mediate the locomotion activating effect of NPS, as pharmacological inhibition or genetic deletion of CRF_1_ receptors block the effect of NPS on locomotion [18]. The 22 kHz USV eliciting effect of NPS may thus be related to the activation of the central CRF-system. Indeed, CRF seems to facilitate 22 kHz USV production in somewhat stressful conditions [53]. For example, in adult rats, pretreatment with CRF_1_ antagonist NBI 35965 administered into the dorsal raphe nucleus selectively attenuated the 22 kHz USVs induced by foot-shock stressor [54], and in chronically foot-shocked rats, CRF treatment increased conditioned vocalization, thus suggesting a modulatory role for CRF in 22 kHz USV’s [55]. However, i.c.v. CRF was shown not to modify the number of 22 kHz calls on its own [55]. 

The long 22 kHz type of vocalizations can be pharmacologically induced by direct cholinergic stimulation of the brain at multiple sites [34,56,57]. There is evidence of co-localisation of NPS with acetylcholine [58], so further studies should address the possible cholinergic mediation of NPS-elicited alarm calls. The behavioural role of short 22 kHz vocalizations as compared to long calls is not entirely clear, but it has been suggested that short versus long 22 kHz USVs express different states: negative emotional states associated with an “internal” discontent (i.e., without an external danger or threat) could be expressed in short USVs, while the external danger is expressed by long USVs [34]. Further studies employing NPS, CRF_1_ and acetylcholine receptor antagonists are needed to specify the mechanism behind the production of different types of 22 kHz USVs. 

Acute NPS treatment elicited similar number of 22 kHz USVs in HE- and LE-rats. Nevertheless, when the 22 kHz USVs were analysed with regard to their duration, average peak frequency, and loudness, some remarkable differences between HE- and LE-rats were revealed. In LE-rats the long 22 kHz USVs were emitted at significantly lower average peak frequencies and were louder during the first 15 min after i.c.v. administration of NPS as if indicative of a more pronounced emotional state. Previously, dose-dependent effects of carbachol, a muscarinic agonist, on acoustic characteristics of 22 kHz USVs have been demonstrated, while the loudness of the 22 kHz USVs was increased with the increasing dosage of carbachol [57].

It has been suggested that i.c.v. injections of NPS may have rewarding effect, can facilitate seeking behaviour, and elicit reinforcement, although these effects seem not robust. In addition, unlike drugs of abuse, NPS appears to lack the ability to sensitise seeking-related behavior [59]. NPS has been shown to promote dopaminergic activity in the brain, which in turn is known to induce 50 kHz vocalizations, but in this experiment, NPS neither induced 50 kHz calls nor attenuated the novelty-associated USVs. Previously, the compounds that have induced 22 kHz calls have also attenuated the 50 kHz USVs. For example, naloxone increased the emission of 22 kHz USVs and concurrently decreased the emission of 50 kHz calls [27]. Thus, the USV profile after NPS is unique. Interestingly, among NPS-induced 22 kHz USVs some had the 50 kHz component. The conditions of production of such mixed calls require further examination. 

Our previous studies have shown that the HE/LE phenotypes show differential resiliency to a variety of interventions [44,46,60]. In the present study and in agreement with our previous results, the HE-rats were more active in the battery of behavioural tests after the i.c.v. procedure, independent of NPS/vehicle treatment. In LE-rats, NPS promoted risk-assessment behaviour in terms of increased stretch-attend postures towards the open area in the exploration box test and had an anxiolytic effect in the elevated zero-maze. In contrast, NPS elicited mainly locomotor activation in the HE-rats. In the elevated zero-maze, NPS facilitated entries into the open quadrants and promoted line-crossings there in the HE-rats, and this locomotion-activating effect was also seen during the first 5 min in the novel housing cage. 

NPS did not modify behavior in the light-dark test in either HE- or LE-rats. The light-dark box test was carried out as the third test (approx. 55 min after the i.c.v. injection) and the light compartment was lit brighter than in the other tests, thus the test environment was novel and probably more anxiogenic to the rats. The effect of NPS could be worn off by this time; however, NPS was able to stimulate locomotion in the HE-rats (both line crossing and rearings) in the subsequent (last) behavioural test (the large rat housing cage), as well as to bring about increased number of long 22 kHz and mixed-type calls there. 

Thus it seems that NPS had an anxiolytic effect in LE-rats in a novel and low-aversive environment and mainly increased mobility in HE-rats. Previously, some indications about inter-individual variability in response to NPS have been made. The reduction in anxiety-related behaviour in the plus-maze after i.c.v. NPS was particularly pronounced in Flinders Sensitive Line and Sprague–Dawley male rats as compared to the Flinders Resistant Line [61]. Similarly, NPS was reported to increase the proportion of time spent by anxious HAB-rats in the open arms of the elevated plus-maze, and also to promote their locomotor activity, while in non-selectively bred Wistars rats this activity-enhancing effect was absent [62]. Of course, certain limitations of the present study prevent excessive generalisation: First, the same battery of behavioural tests carried out in the same order was applied to every rat, thus we cannot rule out carry-over effects. Second, NPS was administered at a single dose.

Conclusively, neuropeptide S was found to elicit a massive burst of 22 kHz ultrasonic vocalizations that suggests that the stimulant effect of NPS has an aversive component, previously not reported for this behaviourally activating neuropeptide. The anxiolytic-like and activating effects of NPS were not accompanied by 50 kHz USVs, the indication of positive affect, but varied due to persistent inter-individual differences, a finding well in line with the above discussed genetic studies in humans. Higher anxiety levels of the low exploratory phenotype were also revealed in the USV profile under stimulation by NPS. Thus, NPS appears to be a potent regulator of affective states that vary by individual adaptive strategies. 

## 4. Materials and Methods

### 4.1. Animals 

Male Wistar rats (Harlan Laboratories, Venray, The Netherlands) weighing 240–340 g at the start of the experiment were used. The rats were group-housed by four in standard transparent polypropylene cages with wood-chip bedding in a colony room maintained at 21 ± 1 °C on a 12 h light/dark cycle (lights on at 08:00 h). Tap water and chow pellets (diet R70, Lactamin AB, Kimstad, Sweden) were available *ad libitum*. The surgery and behavioural experiments were performed in separate rooms within the animal house between 11:00 and 18:00 h. The experimental protocol was approved by the Animal Experimentation Committee at the Estonian Ministry of Agriculture (18 August 2014, no. 38). 

### 4.2. General Procedure

Rats with the highest (HE: high exploring rats) and lowest exploratory (LE: low exploring rats) activity were drawn from a larger pool of rats (*n* = 100) based on the sum of the exploratory events on the second exposure to the exploration box test [44] (see Section 4.6.1); Male rats had a strongly bimodal distribution in this test. The average sum of exploratory events (line crossing, rearing, object investigation) was 230 ± 5 and 4.9 ± 1.7 for HE and LE animals, respectively (F(1,46) = 1818, *p* < 0.001)). Based on observation of about two thousand animals, we categorise as LE those rats that have <50 events and as HE, those with >100. The lowest individual value for HE was 186 and the highest for LE was 30 in this study.

Next, rats were implanted with the i.c.v. guide cannula. Postoperatively, the rats were weighed and handled daily for 6 days, and the dummy cannula was removed twice during this period in order to habituate the animals to the procedure of intracerebral injection, thereby minimizing the stress reaction on the day of behavioural experiments.

On the experiment day (7 days from surgery), immediately after the i.c.v. injection of NPS/Vehicle, the rats were returned to their individual home cage in the dimly lit experiment room, where their ultrasonic vocalizations were registered for 30 min. After that, the behavioural tests were carried out in a fixed order as follows: elevated zero maze (5 min), exploration box test (15 min), light-dark box (5 min). Finally, approx. 1 h after the i.c.v. infusion, the rats were placed into a novel standard housing cage, and their USVs and locomotor activity was recorded for 15 min. At the end of the experiment day, the rats were given an i.c.v injection of 5 µL of methyl green solution (AppliChem GmbH, Darmstadt, Germany) to verify the injection placement and decapitated; the brains were removed, immediately frozen on dry ice, and kept at −80 °C. The brains were sectioned on a cryostatic microtome (Microm GmbH, Walldorf, Germany), and intracerebroventricular colouring was estimated. Only rats with extensive green intracerebroventricular colouring were used for the analysis (n=36).

### 4.3. Drugs

Rat NPS (Ser-Phe-Arg-Asn-Gly-Val-Gly-Ser-Gly-Val-Lys-Lys-Thr-Ser-Phe-Arg-Arg-Ala-Lys-Gln) (Sigma-Aldrich Co, St. Louis, MO, USA) was diluted with Ringer’s solution (140 mM NaCl, 4 mM KCl, 1.2 mM CaCl_2_, 1.0 mM MgCl_2_, 1.0 mM Na_2_HPO_4_, 0.2 mM NaH_2_PO_4_; pH 7.2) to a final concentration of 1.0 nmol/5 µL [61]. Vehicle animals received a 5 µL infusion of Ringer’s solution. The aliquots of NPS were stored at −80 °C. All drugs were kept on ice during the experimental procedures. 

### 4.4. Stereotaxic Surgery

Prior to surgery the rats were weighed, housed individually and anaesthetised with combined solution of ketamine (Bioketan; Vetoquinol Biowet Sp.z.o.o., Gorzów Wielkopolski, Poland) and medetomidine (Domitor, Orion Corporation, Espoo, Finland) (45 mg/kg and 0.2 mg/kg i.p., respectively). The anaesthetised animal was mounted in a stereotaxic frame (David Kopf Instruments, Tujunga, CA, USA) and kept on a heating pad. A 13 mm long 22-gauge i.c.v. guide cannula was aimed at the right lateral ventricle (AP +0.6, ML −1.3, DV −4.0, according to the brain atlas of Paxinos and Watson [63]), and was fixed on the skull with two stainless steel screws and dental cement. A dummy was inserted into the guide cannula. Following surgery, the animals were injected with ampicillin (100 mg/kg s.c., Alfasan International BV, JA Woerden, The Netherlands), butorfanol (Butomidor, 2 mg/kg s.c., Richter Pharma AG, Wels, Austria), carprofen (Rimadyl 0.2 mg/kg s.c., Pfizer Animal Health Ma Eeig, Sandwich, UK) and 2.5 mL sterile saline i.p. and returned to their home cage for recovery.

### 4.5. I.c.v. Procedure

For the infusion, a 14 mm-long 27 gauge cannula was inserted into a 2 cm polyamine tubing (0.38 mm inner diameter, 1.09 mm outer diameter) connected to 8 cm long flexible FEP-tubing (0.12 mm inner diameter, 0.68 mm outer diameter, AgnTho’s AB, Lindigö, Sweden), which in turn was attached to a 10 µL Hamilton syringe (Hamilton Bonaduz AG, Bonaduz, Switzerland). The infusion system was filled with either 5 µL NPS or vehicle, which was then injected over a period of 1 min and left in place for 30 s to allow diffusion. Immediately after the injection, the rats were returned to their home cage.

### 4.6. Behavioural Experiments

#### 4.6.1. Exploration Box Test

**Figure 11:**
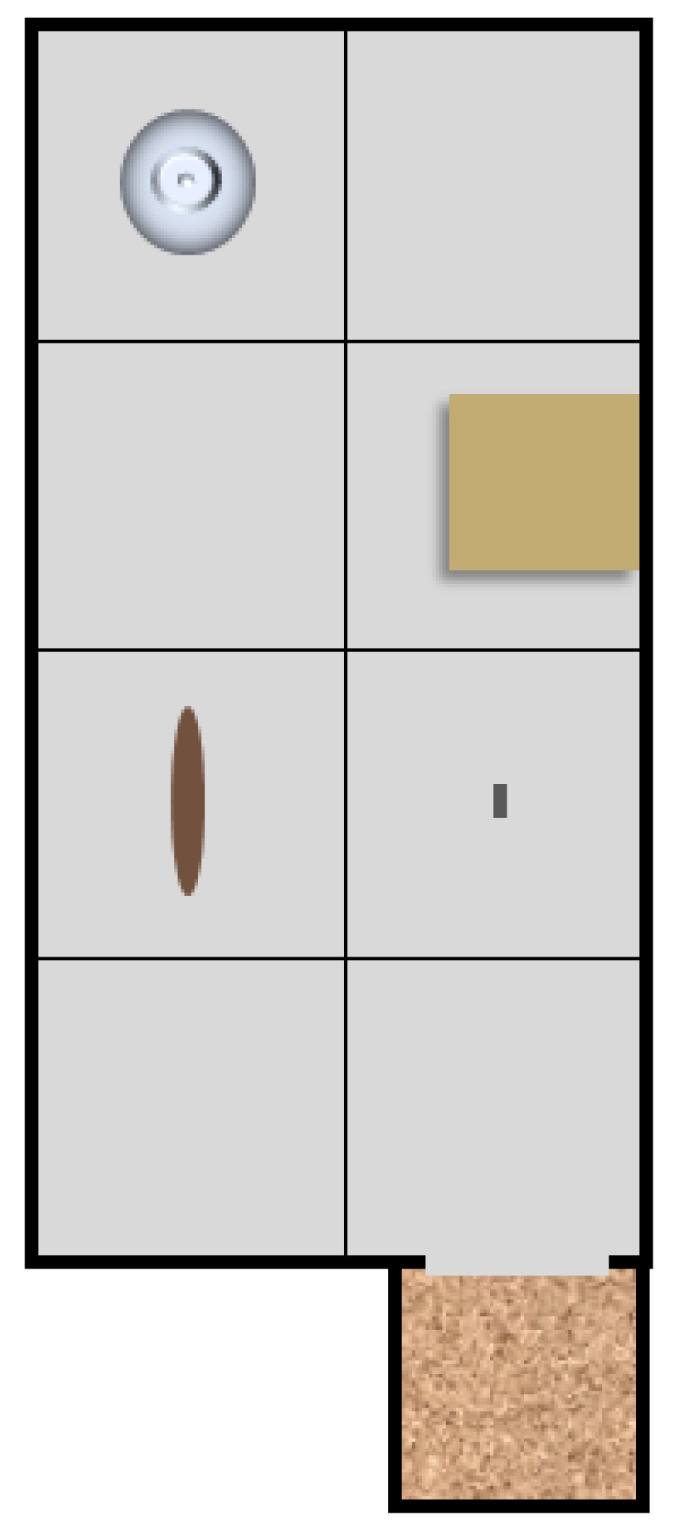
Schematic layout of the exploration box test. Objects from upper left quadrant clockwise: a glass jar, a cardboard box, a food pellet, a wooden handle. In control, untreated conditions, the novel objects are investigated to a rather equal extent, while the food pellet is ignored.

The exploration box [43] was made of metal and comprised a 50 cm × 100 cm open area (height of side walls 40 cm) and a 20 cm × 20 cm × 20 cm small compartment attached to one of the shorter sides of the open area. The open area was divided into eight squares of equal size, and four objects, three novel (a glass jar, a cardboard box, and a wooden handle) and one familiar (a food pellet) were placed in certain squares so that the locations of the objects remained the same throughout the experiment (Figure 11). The floor of the small compartment was covered with wood shavings. The open area was directly accessible from the small chamber through an opening (20 cm × 20 cm). The exploration test was initiated by placing the rat into the small compartment, which was then covered with a lid for the duration of the test. The following parameters were registered by an observer: (1) stretch-attend postures (SAP) toward the open area (forepaws in the open arena, elongated body posture, intensive sniffing, but the rat retreats into the enclosed area), (2) latency to enter the open area with all four paws (s), (3) entries into the open area, (4) time spent exploring on the open area (s), (5) line crossings, (6) rearings (rat on two hind paws, at an angle of at least 45°), and (7) number of investigations of the three unfamiliar objects. To provide an index of exploration considering the elements of both inquisitive and inspective exploration, the scores of line crossings, rearings and object investigations were summed for each animal and thus, (8) the sum of exploratory activity was obtained. After each animal, the open area was wiped clean with a wet tissue. A single test session lasted 15 min and was carried out under dim light conditions (4–5 lux in the open area). 

During pre-selection, all animals were exposed to the exploration box on two consecutive days. The classification of rats into groups of high or low explorers (HE and LE, respecitively) was based on the sum of the exploratory events on the second exposure to the exploration box test [44]. The third exploration box test was carried out 40 min after the i.c.v. infusion. 

#### 4.6.2. Elevated Zero-Maze Test

The elevated zero-maze test [64,65] was conducted as previously described [60]. An elevated annular platform (outer Ø 105 cm, width 10 cm, 72 cm above the floor) was equally divided into two opposing enclosed quadrants (height of the walls 28 cm) that were connected by open quadrants (height of the edge 1 cm, 5–11 lux in the open part). The open quadrants were also divided into three equidistant parts to quantify the locomotor activity. Test rat was placed at the outset of one of the closed quadrants and was observed at a distance for 5 min. (1) SAPs toward the open quadrants, (2) latency to enter the open quadrant with all four paws (s), (3) entries into the open quadrants, (4) time spent (s), and (5) line-crossings in the open quadrants were scored. The apparatus was cleaned with moist tissue after every rat. 

#### 4.6.3. Light-Dark Box Test

The light-dark box test was modified from Henninger et al. [66]. A metal box measuring 30 cm × 60 cm × 40 cm was divided into two equal-sized compartments (30 cm × 30 cm). The dark compartment had a removable cover; the light compartment was open at the top and lighted from above (on average 110 lux). The floor of the light compartment was divided into four 15 cm × 15 cm squares. An opening of 10 cm × 10 cm in the partition wall enabled the rat to alternate between the compartments. The rat was placed at the opening facing the dark compartment, and for the next 5 min, the following measures were registered in the light compartment: (1) SAPs toward the light compartment, (2) latency to enter with all four paws (s), (3) entries into, (4) time (s), (5) line crossings, and (6) rearings. The apparatus was wiped clean after every rat. 

#### 4.6.4. Locomotor Activity in a Novel Large Rat Housing Cage

Under dim conditions (5 lux), a standard group-housing cage that was novel to the rat was used. The cage floor (55 cm × 33 cm) was covered with wood chips, and the cage (height 19 cm) was covered with a flat wire-mesh lid. The rat was initially placed to a corner of the cage and its activity was recorded by a digital video camera. Simultaneously, the USVs emitted were recorded.

Both vertical (rearings) and horizontal locomotor activity (line crossings) were scored from the digital video recordings by an observer blind to the experimental conditions in 3 × 5 min time bins. In order to estimate horizontal locomotor activity, the floor of the housing cage was divided into 6 equal-sized squares, and line crossings with all four paws were counted.

### 4.7. Recording and Scoring of USVs

An ultrasound microphone (Avisoft Ultra Sound Gate 116–200, Avisoft Bioacoustics, Berlin, Germany) was located about 30 cm from the floor of the cage, recording USVs with a sampling rate of 300 kHz in 16-bit format on a computer hard drive [67]. The files were later analysed with Avisoft SASLab Pro (Avisoft Bioacoustics, Berlin, Germany) software. Spectrograms were created using the Fast Fourier Transform algorithm (1024 FFT length, 75% frame, Hamming window, and 75% time window overlap). USVs were manually marked and saved on the spectrogram by an observer blind to experimental conditions. The number and duration (offset of the signal minus the onset, in ms) of the USVs were measured automatically using SASLab Pro and average peak frequency (the average frequency at the onset and offset of the call, in Hz) and average peak amplitude (the average frequency at the onset and offset of the call, intensity in decibels (dB)) were calculated for each of the USVs. 

The following categories of USVs were distinguished: 50 kHz (USVs with frequencies over 35 kHz, including flat, frequency-modulated and trill calls); short 22 kHz (<300 ms; <35 kHz) and long 22 kHz (≥300 ms [33], <35 kHz), and combined 22/50 kHz vocalizations where both components were present. 

### 4.8. Statistical Analysis

Statistical analysis was performed using StatView 5.0. (SAS Institute Inc., Cary, NC, USA), Statistica 8.0. (StatSoft Inc., Tulsa, OK, USA) and R 4.0.2 (R Foundation for Statistical Computing, Vienna, Austria) software. Data from the behavioural tests and the number of ultrasonic vocalizations were analysed using two-factor ANOVA, with *Activity* (HE vs. LE) and *NPS* (vehicle vs. NPS) as independent variables, and repeated measures factor *Time* was added where appropriate. Group differences after significant ANOVAs were measured by *post hoc* Fisher’s Protected Least Significant Difference (PLSD) test. For correlations, Pearson’s correlation coefficient was used. 

In order to analyse whether the features of NPS-induced long and short 22 kHz USVs would be different in HE- and LE-rats during the 30-min period studied, duration, average peak frequency, and average peak amplitude of the USVs were each modelled as predicted by the exploratory phenotype and time-point using two modelling frameworks: linear mixed-effects models (LMM) were fitted with “nlme” (version 3.1-148), generalised additive models (GAM) were fitted with “mgcv” package (version 1.8-31). The data were complex, as USVs were nested within time bins which were further nested within rats. The non-linear interaction of time with the phenotype was evident from exploratory graphs. Therefore flexible models were chosen where the effect of time by phenotype interaction was modelled by either a third-order (cubic) orthogonal polynomial (LMM) [68] or by a cubic spline (GAM) [69] with fixed effects of phenotype (HE vs. LE) on all time points in both approaches. The hierarchical structure of the data was modelled as time-dependent random effects to account for the correlated structure of the data collected from the same rat. Likelihood ratio tests and Akaike information criterion (AIC) were used to select the best fitting model within each model category. In the case of LMMs, the data were fitted using restricted maximum likelihood (REML) for parameter estimation or using maximum likelihood (ML) estimation for nested model comparisons. First-order autoregressive structure was specified for the residuals for each rat to account for the temporal dependence of measurements. In the case of GAMs, the time-dependent trajectory was modelled by a cubic regression spline with 6 knots. The effect of exploratory phenotype was calculated as a difference spline with regard to the reference spline calculated on HE-rats. The random effects were specified similarly to linear mixed models, but instead of orthogonal polynomials, they were represented by cubic splines. The functional form of the outcome variable was modelled by either Gaussian or Gamma distribution, whichever fit best based on AIC score and diagnostic plots. Final models were fitted using REML.

## Figures and Tables

**Table 1:** Overview of the effects of NPS (1 nmol, i.c.v.) on ultrasonic vocalizations and behaviour depending on the exploration phenotype.

*Measure*	LE	HE
***USVs***		
*Home-cage*	↑ short and long 22 kHz USVs	↑ short and long 22 kHz USVs
	↑ 22/50 kHz USVs	↑ 22/50 kHz USVs
	--- 50 kHz USVs	--- 50 kHz USVs
	↓ average peak frequency in long 22 kHz USVs *	
	↑ average peak amplitude in long 22 kHz USVs *	
*Novel large rat cage*	↑ long 22 kHz USVs	↑ long 22 kHz USVs
	↑ 22/50 kHz USVs	↑ 22/50 kHz USVs
	--- 50 kHz, short 22 kHz USVs	--- 50 kHz, short 22 kHz USVs
***Behavioural tests***		
*Exploration box*	↑ stretch-attend postures	↓ time in the open area while no change in exploratory activity
*Elevated 0-maze*	↓ latency to enter	
	↑ time in the open quadrants	
	↑ entries	↑ entries
	↑ line crossings	↑ line crossings
*Light-dark box*	---	---
*Novel large rat cage*	---	↑ rearings and line crossings during first 5 min

↑ denotes increase; ↓ denotes decrease; --- denotes no effect; *: as compared to HE-rats. LE: low exploring rats; HE: high exploring rats; USVs: ultrasonic vocalizations.

## Data Availability

The data files are available upon reasonable request from the corresponding author.

## References

[1] Xu Y.-L.L., Reinscheid R.K., Huitron-Resendiz S., Clark S.D., Wang Z., Lin S.H., Brucher F.A., Zeng J., Ly N.K., Henriksen S.J. (2004). Neuropeptide S: A neuropeptide promoting arousal and anxiolytic-like effects. Neuron.

[2] Xu Y.-L., Gall C.M., Jackson V.R., Civelli O., Reinscheid R.K. (2007). Distribution of neuropeptide S receptor mRNA and neurochemical characteristics of neuropeptide S-expressing neurons in the rat brain. J. Comp. Neurol..

[3] Rizzi A., Vergura R., Marzola G., Ruzza C., Guerrini R., Salvadori S., Regoli D., Calo G. (2008). Neuropeptide S is a stimulatory anxiolytic agent: A behavioural study in mice. Br. J. Pharmacol..

[4] Pulga A., Ruzza C., Rizzi A., Guerrini R., Calo G. (2012). Anxiolytic- and panicolytic-like effects of Neuropeptide S in the mouse elevated T-maze. Eur. J. Neurosci..

[5] Ensho T., Nakahara K., Suzuki Y., Murakami N. (2017). Neuropeptide S increases motor activity and thermogenesis in the rat through sympathetic activation. Neuropeptides.

[6] Vitale G., Filaferro M., Ruggieri V., Pennella S., Frigeri C., Rizzi A., Guerrini R., Calò G. (2008). Anxiolytic-like effect of neuropeptide S in the rat defensive burying. Peptides.

[7] Leonard S.K., Dwyer J.M., Sukoff Rizzo S.J., Platt B., Logue S.F., Neal S.J., Malberg J.E., Beyer C.E., Schechter L.E., Rosenzweig-Lipson S. (2008). Pharmacology of neuropeptide S in mice: Therapeutic relevance to anxiety disorders. Psychopharmacology.

[8] Reinscheid R.K., Xu Y.L., Okamura N., Zeng J., Chung S., Pai R., Wang Z., Civelli O. (2005). Pharmacological characterization of human and murine neuropeptide S receptor variants. J. Pharmacol. Exp. Ther..

[9] Domschke K., Reif A., Weber H., Richter J., Hohoff C., Ohrmann P., Pedersen A., Bauer J., Suslow T., Kugel H. (2011). Neuropeptide S receptor gene—Converging evidence for a role in panic disorder. Mol. Psychiatry.

[10] Dannlowski U., Kugel H., Franke F., Stuhrmann A., Hohoff C., Zwanzger P., Lenzen T., Grotegerd D., Suslow T., Arolt V. (2011). Neuropeptide-S (NPS) receptor genotype modulates basolateral amygdala responsiveness to aversive stimuli. Neuropsychopharmacology.

[11] Laas K., Reif A., Akkermann K., Kiive E., Domschke K., Lesch K.P., Veidebaum T., Harro J. (2014). Interaction of the neuropeptide S receptor gene Asn107Ile variant and environment: Contribution to affective and anxiety disorders, and suicidal behaviour. Int. J. Neuropsychopharmacol..

[12] Laas K., Eensoo D., Paaver M., Lesch K.P., Reif A., Harro J. (2015). Further evidence for the association of the NPSR1 gene A/T polymorphism (Asn107Ile) with impulsivity and hyperactivity. J. Psychopharmacol..

[13] Wang K., Wang J., Zhu C., Yang L., Ren Y., Ruan J., Fan G., Hu J., Xu W., Bi X. (2021). African lungfish genome sheds light on the vertebrate water-to-land transition. Cell.

[14] Reinscheid R.K., Mafessoni F., Lüttjohann A., Jüngling K., Pape H.C., Schulz S. (2021). Neandertal introgression and accumulation of hypomorphic mutations in the neuropeptide S (NPS) system promote attenuated functionality. Peptides.

[15] Ebner K., Rjabokon A., Pape H.C., Singewald N. (2011). Increased in vivo release of neuropeptide S in the amygdala of freely moving rats after local depolarisation and emotional stress. Amino Acids.

[16] Smith K.L., Patterson M., Dhillo W.S., Patel S.R., Semjonous N.M., Gardiner J.V., Ghatei M.A., Bloom S.R. (2006). Neuropeptide S stimulates the hypothalamo-pituitary-adrenal axis and inhibits food intake. Endocrinology.

[17] Jüngling K., Liu X., Lesting J., Coulon P., Sosulina L., Reinscheid R.K., Pape H.C. (2012). Activation of neuropeptide S-expressing neurons in the locus coeruleus by corticotropin-releasing factor. J. Physiol..

[18] Pañeda C., Huitron-Resendiz S., Frago L.M., Chowen J.A., Picetti R., De Lecea L., Roberts A.J. (2009). Neuropeptide S reinstates cocaine-seeking behavior and increases locomotor activity through corticotropin-releasing factor receptor 1 in mice. J. Neurosci..

[19] Mochizuki T., Kim J., Sasaki K. (2010). Microinjection of neuropeptide S into the rat ventral tegmental area induces hyperactivity and increases extracellular levels of dopamine metabolites in the nucleus accumbens shell. Peptides.

[20] Si W., Aluisio L., Okamura N., Clark S.D., Fraser I., Sutton S.W., Bonaventure P., Reinscheid R.K. (2010). Neuropeptide S stimulates dopaminergic neurotransmission in the medial prefrontal cortex. J. Neurochem..

[21] Harro J. (2018). Animals, anxiety, and anxiety disorders: How to measure anxiety in rodents and why. Behav. Brain Res..

[22] Panksepp J. (1998). Affective Neuroscience: The Foundations of Human and Animal Emotions.

[23] Brudzynski S.M. (2013). Ethotransmission: Communication of emotional states through ultrasonic vocalization in rats. Curr. Opin. Neurobiol..

[24] Simola N., Granon S. (2019). Ultrasonic vocalizations as a tool in studying emotional states in rodent models of social behavior and brain disease. Neuropharmacology.

[25] Burgdorf J., Panksepp J., Moskal J.R. (2011). Frequency-modulated 50 kHz ultrasonic vocalizations: A tool for uncovering the molecular substrates of positive affect. Neurosci. Biobehav. Rev..

[26] Kroes R.A., Burgdorf J., Otto N.J., Panksepp J., Moskal J.R. (2007). Social defeat, a paradigm of depression in rats that elicits 22-kHz vocalizations, preferentially activates the cholinergic signaling pathway in the periaqueductal gray. Behav. Brain Res..

[27] Burgdorf J., Knutson B., Panksepp J., Shippenberg T.S. (2001). Evaluation of rat ultrasonic vocalizations as predictors of the conditioned aversive effects of drugs. Psychopharmacology.

[28] Blanchard R.J., Weiss S. (1991). Twenty-two kHz alarm cries to presentation of a predator, by laboratory rats living in visible burrow systems. Physiol. Behav..

[29] Sánchez C. (2003). Stress-induced vocalisation in adult animals. A valid model of anxiety?. Eur. J. Pharmacol..

[30] Burgdorf J., Kroes R.A., Moskal J.R., Pfaus J.G., Brudzynski S.M., Panksepp J. (2008). Ultrasonic vocalizations of rats (Rattus norvegicus) during mating, play, and aggression: Behavioral concomitants, relationship to reward, and self-administration of playback. J. Comp. Psychol..

[31] Panksepp J., Burgdorf J. (2000). 50-kHz chirping (laughter?) in response to conditioned and unconditioned tickle-induced reward in rats: Effects of social housing and genetic variables. Behav. Brain Res..

[32] Burgdorf J., Wood P.L., Kroes R.A., Moskal J.R., Panksepp J. (2007). Neurobiology of 50-kHz ultrasonic vocalizations in rats: Electrode mapping, lesion, and pharmacology studies. Behav. Brain Res..

[33] Brudzynski S.M., Bihari F., Ociepa D., Fu X.W. (1993). Analysis of 22 kHz ultrasonic vocalization in laboratory rats: Long and short calls. Physiol. Behav..

[34] Brudzynski S.M. (2015). Pharmacology of ultrasonic vocalizations in adult rats: Significance, call classification and neural substrate. Curr. Neuropharmacol..

[35] Brudzynski S.M., Iku A., Harness neé Savoy A. (2011). Activity of cholinergic neurons in the laterodorsal tegmental nucleus during emission of 22kHz vocalization in rats. Behav. Brain Res..

[36] Burgdorf J., Knutson B., Panksepp J., Ikemoto S. (2001). Nucleus accumbens amphetamine microinjections unconditionally elicit 50-kHz ultrasonic vocalizations in rats. Behav. Neurosci..

[37] Brudzynski S.M. (2001). Pharmacological and behavioral characteristics of 22 kHz alarm calls in rats. Neurosci. Biobehav. Rev..

[38] Ahrens A.M., Ma S.T., Maier E.Y., Duvauchelle C.L., Schallert T. (2009). Repeated intravenous amphetamine exposure: Rapid and persistent sensitization of 50-kHz ultrasonic trill calls in rats. Behav. Brain Res..

[39] Simola N., Fenu S., Costa G., Pinna A., Plumitallo A., Morelli M. (2012). Pharmacological characterization of 50-kHz ultrasonic vocalizations in rats: Comparison of the effects of different psychoactive drugs and relevance in drug-induced reward. Neuropharmacology.

[40] Harro J. (2010). Inter-individual differences in neurobiology as vulnerability factors for affective disorders: Implications for psychopharmacology. Pharmacol. Ther..

[41] Gould T.D., Gottesman I.I. (2006). Psychiatric endophenotypes and the development of valid animal models. Genes Brain Behav..

[42] Montgomery K.C. (1955). The relation between fear induced by novel stimulation and exploratory drive. J. Comp. Physiol. Psychol..

[43] Otter M.H., Matto V., Sõukand R., Skrebuhhova T., Allikmets L., Harro J. (1997). Characterization of rat exploratory behavior using the exploration box test. Methods Find. Exp. Clin. Pharmacol..

[44] Mällo T., Alttoa A., Kõiv K., Tõnissaar M., Eller M., Harro J. (2007). Rats with persistently low or high exploratory activity: Behaviour in tests of anxiety and depression, and extracellular levels of dopamine. Behav. Brain Res..

[45] Alttoa A., Kõiv K., Hinsley T.A., Brass A., Harro J. (2010). Differential gene expression in a rat model of depression based on persistent differences in exploratory activity. Eur. Neuropsychopharmacol..

[46] Alttoa A., Kõiv K., Eller M., Uustare A., Rinken A., Harro J. (2005). Effects of low dose N-(2-chloroethyl)-N-ethyl-2-bromobenzylamine administration on exploratory and amphetamine-induced behavior and dopamine D2 receptor function in rats with high or low exploratory activity. Neuroscience.

[47] Alttoa A., Seeman P., Kõiv K., Eller M., Harro J. (2009). Rats with persistently high exploratory activity have both higher extracellular dopamine levels and higher proportion of D2 High receptors in the striatum. Synapse.

[48] O’Leary A., Kõiv K., Raudkivi K., Harro J. (2016). Antidepressants differentially affect striatal amphetamine-stimulated dopamine and serotonin release in rats with high and low novelty-oriented behaviour. Pharmacol. Res..

[49] Brudzynski S.M. (2005). Principles of rat communication: Quantitative parameters of ultrasonic calls in rats. Behav. Genet..

[50] Basken J.N., Connor N.P., Ciucci M.R. (2012). Effect of aging on ultrasonic vocalizations and laryngeal sensorimotor neurons in rats. Exp. Brain Res..

[51] Zhu H., Mingler M.K., McBride M.L., Murphy A.J., Valenzuela D.M., Yancopoulos G.D., Williams M.T., Vorhees C.V., Rothenberg M.E. (2010). Abnormal response to stress and impaired NPS-induced hyperlocomotion, anxiolytic effect and corticosterone increase in mice lacking NPSR1. Psychoneuroendocrinology.

[52] Simola N., Paci E., Serra M., Costa G., Morelli M. (2018). Modulation of Rat 50-kHz Ultrasonic Vocalizations by Glucocorticoid Signaling: Possible Relevance to Reward and Motivation. Int. J. Neuropsychopharmacol..

[53] Ise S., Nagano N., Okuda S., Ohta H. (2008). Corticotropin-releasing factor modulates maternal separation-induced ultrasonic vocalization in rat pups via activation of CRF1 receptor. Brain Res..

[54] Li C., McCloskey N., Phillips J., Simmons S.J., Kirby L.G. (2020). CRF-5-HT interactions in the dorsal raphe nucleus and motivation for stress-induced opioid reinstatement. Psychopharmacology.

[55] Swiergiel A.H., Zhou Y., Dunn A.J. (2007). Effects of chronic footshock, restraint and corticotropin-releasing factor on freezing, ultrasonic vocalization and forced swim behavior in rats. Behav. Brain Res..

[56] Brudzynski S.M., Ociepa D., Bihari F. (1991). Comparison between cholinergically and naturally induced ultrasonic vocalization in the rat. J. Psychiatry Neurosci..

[57] Brudzynski S.M. (1994). Ultrasonic vocalization induced by intracerebral carbachol in rats: Localization and a dose-response study. Behav. Brain Res..

[58] Reinscheid R.K. (2008). Neuropeptide S: Anatomy, pharmacology, genetics and physiological functions. Results Probl. Cell Differ..

[59] Cao J., De Lecea L., Ikemoto S. (2011). Intraventricular administration of neuropeptide S has reward-like effects. Eur. J. Pharmacol..

[60] Matrov D., Kõiv K., Kanarik M., Peet K., Raudkivi K., Harro J. (2016). Middle-range exploratory activity in adult rats suggests higher resilience to chronic social defeat. Acta Neuropsychiatr..

[61] Wegener G., Finger B.C., Elfving B., Keller K., Liebenberg N., Fischer C.W., Singewald N., Slattery D.A., Neumann I.D., Mathé A.A. (2012). Neuropeptide S alters anxiety, but not depression-like behaviour in Flinders Sensitive Line rats: A genetic animal model of depression. Int. J. Neuropsychopharmacol..

[62] Slattery D.A., Naik R.R., Grund T., Yen Y.C., Sartori S.B., Füchsl A., Finger B.C., Elfving B., Nordemann U., Guerrini R. (2015). Selective breeding for high anxiety introduces a synonymous SNP that increases Neuropeptide S receptor activity. J. Neurosci..

[63] Paxinos G., Watson C. (2007). The Rat Brain in Stereotaxic Coordinates.

[64] Shepherd J.K., Grewal S.S., Fletcher A., Bill D.J., Dourish C.T. (1994). Behavioural and pharmacological characterisation of the elevated “zero-maze” as an animal model of anxiety. Psychopharmacology.

[65] Matto V., Harro J., Allikmets L. (1997). The effects of cholecystokinin A and B receptor antagonists on exploratory behaviour in the elevated zero-maze in rat. Neuropharmacology.

[66] Henniger M.S.H., Ohl F., Hölter S.M., Weißenbacher P., Toschi N., Lörscher P., Wigger A., Spanagel R., Landgraf R. (2000). Unconditioned anxiety and social behaviour in two rat lines selectively bred for high and low anxiety-related behaviour. Behav. Brain Res..

[67] Kõiv K., Tiitsaar K., Laugus K., Harro J. (2021). Extracellular Dopamine Levels in Nucleus Accumbens after Chronic Stress in Rats with Persistently High vs. Low 50-kHz Ultrasonic Vocalization Response. Brain Sci..

[68] Mirman D. (2014). Growth Curve Analysis and Visualization Using R.

[69] Wood S.N. (2017). Generalized Additive Models: An Introduction with R.

